# A hybrid approach to enhance HbA1c prediction accuracy while minimizing the number of associated predictors: A case-control study in Saudi Arabia

**DOI:** 10.1371/journal.pone.0326315

**Published:** 2025-06-17

**Authors:** Faten Al-hussein, Mali Abdollahian, Laleh Tafakori, Khalid Al-Shali

**Affiliations:** 1 School of Science, RMIT University, Melbourne, Victoria, Australia; 2 Department of Mathematics and Statistics, College of Sciences, University of Jeddah, Jeddah, Saudi Arabia; 3 Department of Medicine, King Abdulaziz University Hospital, Jeddah, Saudi Arabia; University of Lagos Faculty of Engineering, NIGERIA

## Abstract

Type 2 diabetes (T2D) is considered a significant global health concern. Hemoglobin A1c level (HbA1c) is recognized as the most reliable indicator for its diagnosis. Genetic, family, environmental, and health behaviors are the factors associated with the disease. T2D is linked to substantial economic costs and human suffering, making it a primary concern for health planners, physicians, and those living with the disease. Saudi Arabia currently ranks seventh worldwide in terms of prevalence rate. Despite this high rate, the country lacks focused research on T2D. This study aims to develop hybrid prediction models that integrate the strengths of multiple algorithms to enhance HbA1c prediction accuracy while minimising the number of significant Key Performance Indicators (KPIs). The proposed model can help healthcare practitioners diagnose T2D at an early stage. Analyses were conducted in a case-control study in Saudi Arabia involving cases (patients with HbA1c levels ≥ 6.5) and controls with normal HbA1c levels (< 6.5). Medical records from 3,000 King Abdulaziz University Hospital patients containing demographic, lifestyle, and lipid profile data were used to develop the models. For the first time, we utilized recommended machine learning algorithms to develop hybrid prediction models to reduce the number of significant KPIs while enhancing HbA1c prediction accuracy. The hybrid model combining Random Forest (RF) and Logistic Regression (LR) with only 4 out of 10 KPIs outperformed other models with an accuracy of 0.93, precision of 0.95, recall of 0.90, F-score of 0.92, an AUC of 0.88, and Gini index of 0.76. The significant variables identified by the model through backward elimination are age, body mass index (BMI), triglycerides (TG), and high-density lipoprotein (HDL). The proposed model helps healthcare providers identify patients at risk of T2D by monitoring fewer key predictors of HbA1c levels, enhancing early intervention strategies for managing diabetes in Saudi Arabia.

## 1. Introduction

Type 2 Diabetes (T2D) is a condition characterized by consistently high blood sugar levels and has become one of the world’s most significant health issues [1]. T2D is a metabolic condition characterised by high blood sugar levels caused by either inadequate insulin synthesis or reduced insulin release by pancreatic β-cells [2]. According to the International Diabetes Federation (IDF) Atlas (2021), 10.5% of persons aged 20–79 have diabetes, with nearly half being ignorant of the diagnosis. Projections for 2045 show that roughly 783 million individuals, or one in eight, will be living with diabetes, indicating a 46% rise, with the ailment accounting for nearly 6.7 million deaths globally in 2021 [3,4]. Diabetes is anticipated to be the 7^th^ greatest cause of death globally by 2030, as reported by the World Health Organisation (WHO) [5].

The incidence of diabetes is projected to be elevated across the Middle East and North Africa (MENA), most likely due to increasing income per person, financial development, urban growth, and significant changes in lifestyle leading to reduced physical activity and higher obesity levels [6,7]. Regional analysis indicates that the largest increase is projected to occur in Africa (156%), followed by the (MENA) region (110%) [8]. Saudi Arabia is one of the nations with the highest prevalence of T2D in the world [9]. In recent years, the country has seen considerable growth in the prevalence of diabetes, with a large portion of the adult population currently suffering from the disease [10]. By 2030, Saudi Arabia is predicted to be in the top 10 Middle Eastern nations with the highest projected prevalence of diabetes, with a prevalence of 20.8% [11]. In 2021, the adult population of Saudi Arabia was 24,194,300, with 17.7% being affected by diabetes, which amounts to a total of 4,274,100 cases [12].

HbA1c levels are a key measure for identifying the early likelihood of T2D, especially among populations at elevated risk [13,14]. HbA1c is commonly used for diagnosing diabetes because it is more convenient and accurate than fasting plasma glucose (FPG) and the oral glucose tolerance test (OGTT) [15]. An HbA1c reading of 6.5% or higher in red blood cells indicates a diagnosis of diabetes [16,17]. These levels can be affected by a variety of factors, including race, age, weight, diet, and overall health status [18]. According to the WHO (2019), the globally recognized HbA1c threshold value is 6.5% (48 mmol/mol), although this may differ for different populations [16].

Extensive research has been conducted on the comparative performance of various machine learning (ML) models in predicting HbA1c levels, utilizing a diverse set of independent variables. A study explored the application of data mining techniques for predicting and diagnosing blood glucose levels in diabetes. The study used a systematic approach to review various machine learning methods, including artificial neural networks (ANN), k-nearest neighbors (KNN), decision trees (J48) (a combination of decision tree learning and information gain ratio, enhanced with pruning to avoid overfitting and handle missing values), and support vector machine (SVM) all of which demonstrated high accuracy [19]. Moreover, another study analyzing data from 18,844 patients based on six medical variables was used to assess the prediction performance of HbA1c levels using several (ML) models, including multiple linear regression (MLR), multilayer perceptron (MLP), support vector machine (SVM), and random forest (RF). The results showed that MLP outperformed other models [20]. Another study, authors applied machine learning models to classify the prevalence of diabetes based on behavioral risk factors, using 1,272 observations across five key variables: age, obesity, physical inactivity, gender, and smoking. The evaluated approaches included k-nearest neighbors (KNN), linear discriminant analysis (LDA), support vector machine (SVM) with multiple kernels, and neural networks (NN), with the KNN model emerging as the top performer [21].

Some researchers have used supervised methods such as machine learning with the Pima Indian Diabetes dataset (PIDD), which contains 768 records and 12 attributes of patients. The dataset has been used in several studies to predict diabetes by classifying HbA1c levels using various machine learning (ML) algorithms. One study [22] showed that the random forest (RF) model was the most effective in predicting HbA1c levels with an accuracy of 92.26%. Another study [23] reported that the random forest (RF) classifier performed well on the PIDD, achieving an accuracy of 89.86%. Researchers in Bangladesh [24] employed various machine learning models with 17 variables and found that the random forest (RF) model demonstrated the highest performance, achieving an impressive accuracy of 98%. A study conducted in Taiwan on a sample of 647 patients with 15 variables to predict glycemic control of HbA1c levels showed that the random forest (RF) outperformed others, achieving an accuracy of 84% [25]. A study conducted in China used machine learning (ML) algorithms to predict HbA1c levels over three months in T2D patients, utilizing 79 variables and 2,169 cases. The data were processed and analyzed using 16 ML methods. The best model for predicting HbA1c was XGBoost, with an accuracy of 74.6% [26]. Another study was conducted in China and included 2,787 patients with T2D. Initially they used 42 variables, and after dimensionality reduction utilizing the Elastic Network (EN) algorithm, 19 variables were selected for predicting HbA1c levels. The study utilized three main machine learning models: random forest (RF), support vector machine (SVM), and back propagation artificial neural network (BP-ANN). The random forest (RF) model yielded the best results, with an accuracy of 79% [27].

It is well documented that combining machine learning algorithms into hybrid models may further improve prediction accuracy, as these models integrate the strengths of multiple algorithms, allowing them to compensate for each other’s weaknesses, thereby enhancing prediction accuracy and increasing reliability [28]. Building on this, several studies have demonstrated the effectiveness of hybrid models in predicting T2D by combining different algorithms to achieve improved predictive performance.

A study conducted in the US [29] used supervised machine learning models (ML) with 10,000 patient records containing six variables to predict T2D. Among the multiple algorithms applied, the ensemble learning model (EL), combining various machine learning techniques, achieved the highest accuracy of 85%. Moreover, a study in India demonstrated the effectiveness of predicting T2D using a hybrid model, which integrated the K-means clustering algorithm with the C4.5 decision tree algorithm (a combination of decision tree learning and information gain ratio, enhanced with pruning to avoid overfitting), utilising the Pima Indian Diabetes dataset (PIDD). The study employed 10-fold cross-validation to ensure the reliability of the model’s results, achieving an accuracy of 92.38% [30]. Moreover, in [31], a hybrid machine learning approach was used that combined classifiers, including artificial neural networks (ANN), support vector machine (SVM), and k-nearest neighbors (K-NN). The results showed that the ensemble approach outperformed individual classifiers, achieving an accuracy of 98.60%. Another study developed a hybrid model using machine learning algorithms, where logistic regression, decision tree, and random forests were integrated to predict T2D. The model reached an accuracy of 99.34% [32].

Furthermore, a study in Bangladesh used a dataset containing 1,000 patients and 10 variables. Researchers employed hybrid machine learning algorithms, combining support vector machine (SVM), decision tree (DT), and random forest (RF) to predict diabetes. The model achieved an accuracy of 90.1% [33]. A study in Saudi Arabia revealed the application of hybrid models for predicting and diagnosing diabetes, which include different models from the same class or various classes. Specifically, the k-means algorithm is used for clustering and pattern extraction, while the decision tree algorithm (C4.5) is employed for classification. The hybrid methods have shown better results than individual models, particularly in classification accuracy. However, the study identified a lack of widespread application of hybrid models and emphasized the underutilization of modern hybrid models’ capabilities compared to using an individual model [19].

As documented above, combining machine learning algorithms into hybrid models can further improve the accuracy of the prediction. The primary motivation of this paper is to enhance the accuracy of T2D prediction by developing and identifying the best hybrid predictive model with the minimum number of KPIs.

To achieve this, we deployed machine learning methods that have recently been shown to outperform other approaches in predicting T2D, together with the set of recommended key performance indicators (KPIs) to develop hybrid models that fit two models to the same data by combining the prediction power of each model to enhance the accuracy. This is followed by utilising a backward elimination approach to eliminate the least important KPIs and retain the most influential ones. The hybrid models with the smallest number of predictors and the best accuracy performance are identified.

The primary contributions of this study are:

This study will further improve the previous research carried out in Saudi Arabia [19–21] by

(i) expanding the range of variables associated with T2D to cover demographic, lifestyle, and lipid profile data.
(ii) developing hybrid models and utilizing backward elimination approach to improve the prediction accuracy of HbA1C levels while reducing the number of significant demographic, lifestyle, and lipid profile predictors associated with T2D.
(iii) the results of the research will assist healthcare professionals in more accurately identifying patients at risk of developing T2D by monitoring smaller number of KPIs.

## 2. Materials and methodology

For this study, the dataset analysis process and the proposed methodology for predicting HbA1c levels were based on the WHO criteria, where patients with HbA1c readings above or equal 6.5% are considered the case group, and those with readings below 6.5% are classified as the control group [34].

### 2.1. Case-control population and sample size

De-identified data from the medical records of 4,526 patients with T2D over the age of 20 at King Abdulaziz University Hospital (KAUH) in Jeddah, Saudi Arabia, between 01/01/2018 and 31/12/2022 are utilised in this research. The data had been previously collected and assessed by medical professionals for reliability. The patients were classified into two groups: the case group with elevated HbA1c levels (≥ 6.5) and the control group with normal or prediabetic HbA1c levels (< 6.5). HbA1c is measured using high-performance liquid chromatography (HPLC) with the D-100 system from Bio-Rad. The analysis is conducted using whole blood samples. Ethics approval was provided by both the RMIT University Human Research Ethics Committee in Australia and the Research Ethics Committee at King Abdulaziz University Hospital (KAUH). This study was retrospective as the analysis was of the observed pre-existing medical records from KAUH; all data were anonymized before analysis. The need for informed consent was waived by the ethics review committee as we have utilised previously obtained de-identified medical data. Access to the data was given on 19/11/2023 after ethics approval was granted. A two-step filtering process was implemented to ensure data quality, as shown in Table 1. In step 1, we removed incomplete variables for 90% of patients. In step 2, we checked the variability of the different variables across the entire cohort; variables with negligible variability among 90% of the cohort were excluded. This filtering reduced the dataset from 35 variables to 18 and decreased the number of patients from 4,526–3,000 patients, with 1,000 cases (HbA1c ≥ 6.5) and 2,000 controls (HbA1c < 6.5).

**Table 1:** Description and frequency of the Features in the case and control groups.

Feature	HbA1c ≥ 6.5 (Cases) (N = 1000)	HbA1c < 6.5 (Control) (N = 2000)
**Gender**		
Female	446 (44.6%)	901 (45.1%)
Male	554 (55.4%)	1099 (54.9%)
**Age**		
20-39	89 (8.9%)	165 (8.3%)
40-59	459 (45.9%)	584 (29.2%)
60-79	216 (21.6%)	907 (45.3%)
80 and above 99	236 (23.6%)	344 (17.2%)
**BMI**		
Underweight	22 (2.2%)	60 (3.0%)
Normal weight	184 (18.4%)	434 (21.7%)
Overweight	337 (33.7%)	830 (41.5%)
Obese	457 (45.7%)	676 (33.8%)
**Marital Status**		
Married	818 (81.8%)	1575 (78.8%)
Not Married	182 (18.2%)	425 (21.2%)
**Nationality**		
Saudi Arabia	639 (63.9%)	1044 (52.2%)
Other	361 (36.1%)	956 (47.8%)
**Occupation**		
Employed	288 (28.8%)	732 (36.6%)
Not Employed	712 (71.2%)	1268 (63.4%)
**Smoking**		
Yes	869 (86.9%)	1075 (53.7%)
No	131 (13.1%)	925 (46.3%)
**Physical Activity**		
Yes	871 (87.1%)	404 (20.2%)
No	129 (12.9%)	1596 (79.8%)
**Type of Food**		
Healthy	351 (35.1%)	1168 (58.4%)
Non-healthy	649 (64.9%)	832 (41.6%)
**Hypertension**		
Yes	782 (78.2%)	702 (35.1%)
No	218 (21.8%)	1298 (64.9%)
**WBC**		
Low	46 (4.6%)	35 (1.8%)
Good	458 (45.8%)	1911 (95.5%)
High	496 (49.6%)	54 (2.7%)
**HDL**		
Normal	585 (58.5%)	216 (10.8%)
Moderately High	224 (22.4%)	1189 (59.5%)
High	191 (19.1%)	595 (29.7%)
**TC**		
Normal	223 (22.3%)	1408 (70.4%)
Moderately High	206 (20.6%)	403 (20.2%)
High	571 (57.1%)	189 (9.4%)
**TG**		
Normal	198 (19.8%)	1325 (66.2%)
Moderately High	206 (20.6%)	474 (23.7%)
High	596 (59.6%)	201 (10.1%)
**Vitamin D**		
Deficient	478 (47.8%)	96 (4.8%)
Insufficient	231 (23.1%)	429 (21.5%)
Sufficient	291 (29.1%)	1475 (73.7%)
**Ferritin**		
Deficient	581 (58.1%)	101 (5.1%)
Insufficient	230 (23.0%)	1628 (81.4%)
Sufficient	189 (18.9%)	271 (13.5%)
**SBP**		
Low	166 (16.6%)	325 (16.2%)
Normal	381 (38.1%)	921 (46.1%)
High	453 (45.3%)	754 (37.7%)
**DBP**		
Low	668 (66.8%)	336 (16.8%)
Normal	181 (18.1%)	1353 (67.6%)
High	151 (15.1%)	311 (15.6%)

BMI: body mass index, HDL: High-Density Lipoprotein, WBC: White Blood Cells, TG: Triglycerides, SBP: Systolic blood pressure, TC: Total Cholesterol, DBP: Diastolic blood pressure.

### 2.2. The Key Performance Indicators (KPIs) classification and case-control comparison

The KPIs are categorized into demographic, lifestyle, and health indicators: the demographic factors include gender, nationality, body mass index (BMI), age, and marital status. Lifestyle factors cover smoking, physical activity, hypertension, type of food and occupation status. The health indicators include total cholesterol (TC), systolic blood pressure (SBP), ferritin level, glycated hemoglobin (HbA1c), diastolic blood pressure (DBP), high-density lipoprotein (HDL), vitamin D, triglycerides (TG), and white blood cell count (WBC).

According to the BMI and WHO guidelines [34,35], there are four BMI categories: underweight (below 18.5 kg/m^2^), normal weight (18.5–24.9 kg/m^2^), overweight (25.0–29.9 kg/m^2^), and obese (30.0 kg/m^2^) and above). HDL levels are categorized as low (under 40 mg/dL for men and under 50 mg/dL for women), adequate (40–59 mg/dL for men and 50–59 mg/dL for women), and high (60 mg/dL or more for both genders) [36]. TG levels are classified as normal (below 149 mg/dL), moderately high (150–199 mg/dL), and high (200 mg/dL and above) [36]. TC levels are grouped into desirable (below 200 mg/dL), moderately high (200–239 mg/dL), and high (240 mg/dL and above) [36]. Vitamin D status is classified as deficient (under 30 ng/mL), insufficient (30–50 ng/mL), and sufficient (50 ng/mL and above) [37]. Normal ferritin levels range from 24–336 µg/L for women and 11–307 µg/L for men, with lower values suggesting iron deficiency and higher values indicating elevation [38].

Table 2 summarises the substantial difference observed between the case and control groups, alongside their corresponding P-values. The case group had an average age of 62 years, whereas the control group’s average age was 70 years. The BMI was notably higher in the case group, averaging 30.8 (P-value < 0.001). The two groups showed significant variation in physical activity (P-value < 0.001). No substantial difference in smoking habits was found between the two groups (P-value > 0.160), which contrasts with prior studies that have emphasized smoking as a key factor contributing to elevated HbA1c levels [24]. Individuals in the case group also showed higher rates of hypertension (P-value < 0.001), with apparent differences in both diastolic and systolic blood pressure. The research found that married persons were more numerous in the control group than the case group (P-value < 0.025), suggesting a possible link between social stability and improved metabolic health. In addition, lipid profiles indicated that the case group had significantly lower levels of HDL (1.24 vs. 1.66; P-value < 0.001), while their harmful lipids, such as total cholesterol (TC) and triglycerides (TG), were substantially larger (P-value < 0.001 for both).

**Table 2:** Comparison of KPIs in case and control group.

Feature	HbA1c≥6.5 (Cases) Mean, (SD)	HbA1c<6.5 (Control) Mean, (SD)	P-value
Gender	0.49 (0.50)	0.51 (0.50)	0.505
Age	62 (15.12)	70(16.13)	<0.001
BMI	30.88 (6.56)	29.09 (6.68)	<0.001
Marital Status	0.79 (0.41)	0.76 (0.43)	0.025
Nationality	0.48 (0.50)	0.49 (0.50)	0.832
Occupation	0.39 (0.49)	0.36 (0.48)	0.015
Smoking	0.49 (0.50)	0.52 (0.50)	0.160
Physical activity	0.85 (0.36)	0.78 (0.42)	<0.001
Type of food	0.48 (0.50)	0.68 (0.47)	<0.001
Hypertension	0.89 (0.32)	0.61 (0.49)	<0.001
SBP	154.35 (45.64)	104 (42.74)	<0.001
DBP	99.89 (31.47)	81.87 (24.55)	<0.001
HDL	1.24 (0.47)	1.66 (0.45)	<0.001
TC	3.55 (2.39)	1.04 (1.42)	<0.001
TG	1.78 (0.73)	1.32 (0.54)	<0.001
Ferritin	252.51 (142.69)	156.52 (102.44)	<0.001
WBC	7.97 (5.51)	9.69 (7.15)	<0.001
Vitamin-D	51.05 (27)	56.52 (29.96)	<0.001

BMI: body mass index, HDL: High-Density Lipoprotein, WBC: White Blood Cells, TG: Triglycerides, SBP: Systolic blood pressure, TC: Total Cholesterol, DBP: Diastolic blood pressure.

### 2.3. Machine learning techniques

Machine learning employs two essential techniques, classification, and clustering, to achieve diverse objectives. Classification, a supervised learning approach, depends on labelled datasets to train models that can categorize unseen data. In contrast, clustering, which belongs to unsupervised learning, groups data into clusters by assessing similarities among elements in each group. The study employed various classification techniques, including logistic regression (LR), decision tree (DT), artificial neural networks (ANNs), random forest (RF), k-nearest neighbors (KNN), support vector machine (SVM), naive bayes (NB), gradient boosting (GB), and extreme gradient boosting (XGBoost), and adaptive boosting (AdaBoost) [39]. For the clustering technique, k-means and the expectation maximization (EM) algorithm were applied [40].

**Classification algorithms**

#### 2.3.1. Logistic Regression (LR).

LR is a statistical method that examines the relationship between predictor variables and a binary response [41]. It is particularly valuable in healthcare, as it allows researchers to estimate the likelihood of outcomes based on key predictors [42]. A significant advantage of LR is its potential to process categorical variables, making it suitable for binary classification tasks. In this model, the probability of success is calculated using a logistic transformation of the independent variables, as illustrated in the equation (1):


$$p_{i}=\frac{exp(\alpha_{0}+\alpha_{1}z_{i1}+\alpha_{2}z_{i2}+\ldots +\alpha_{p}z_{ip})}{1+exp(\alpha_{0}+\alpha_{1}z_{i1}+\alpha_{2}z_{i2}+\ldots +\alpha_{p}z_{ip})}$$ (1)

here, $\pi_{i}$ represents the probability that a sample belongs to a specific category of the binary response variable, often referred to as the ‘success probability’, clearly, $0\leq p_{i}\leq 1$, and the coefficients $\alpha_{0},\alpha_{1},\alpha_{2},\ldots ,\;\alpha_{p}$ correspond to the explanatory variables$z_{i1},\;z_{i2},\;\ldots ,\;z_{ip}.$

#### 2.3.2. Decision Tree (DT).

DT is a model utilized for classification when the target variable is categorical. The tree divides data based on predictor variables into root, internal, and leaf nodes [43]. It grows by minimizing impurity at each node using criteria such as Information Gain Ratio, Gain, and Gini Index [44]. The tree starts with all observations at the root node, and successive splits determine the importance of the predictor variables. The Gini Index selects splitting variables at internal nodes, optimizing the tree structure.

Here, D represents the dataset or the set of observations at a given node in the decision tree. Specifically, it refers to the subset of data points that reach the node during the tree-building process.$C_{i}$ represents the class to which an observation in D may belong and $p_{i}$ represents the likelihood of an observation within D being part of the class $C_{i}$, and it is calculated as shown in equation (2):


$$p_{i}=\;\frac{\left|C_{i},D\right|}{\left|D\right|}.$$ (2)

#### 2.3.3. Random Forest (RF).

RF is a collective technique that uses random selection to generate multiple decision trees from various variables and data subsets [45]. These trees are merged through a majority voting mechanism to create a powerful classifier. Tree depth, the number of predictors per tree, the total number of trees, and the minimal number of observations needed at each leaf node are important factors [46]. To assess performance, the dataset is separated into distinct training and testing sections. The main advantage of this approach is its capacity to assess the significance of variables using metrics such as the Gini Index and model accuracy declines [47].

#### 2.3.4. Support Vector Machine (SVM).

SVM is primarily used in tasks related to classification and regression, making it a type of supervised learning model [48]. It identifies an optimal separating hyperplane, or maximum margin classifier, which maximizes the distance between different classes and minimizes classification errors [48]. SVM utilizes the kernel trick to perform non-linear classification, allowing it to efficiently process complex, high-dimensional datasets [49]. This capability makes SVM particularly valuable in fields requiring precise predictive modeling and risk minimization, such as medical diagnostics.

#### 2.3.5. Naive Bayes classifier (NB).

The NB algorithm is a probabilistic classifier based on Bayes’ Theorem, assuming strong feature independence. This model is effective in applications such as recommendation systems, predictive analytics, and spam filtering due to its simplicity and predictive accuracy [50]. The classification process involves the following four steps:

(i) Model Construction: Deriving sample mean (μ) and variance $\left(\sigma^{2}\right)$ for numeric data and calculating probabilities for categorical data.
(ii) Probability Calculation: Using the formula for each numeric datum, as demonstrated in equation (3):

$$P\left(x_{i}|y\right)=\;\frac{1}{\sqrt{2\pi \sigma^{2}}}\;\exp\left(-\;\frac{{\left(x_{i}-\mu_{y}\right)}^{2}}{2\sigma_{y}^{2}}\right),$$ (3)

where $\mu_{y}$ and $\sigma_{y}^{2}$ are the mean and variance for class y, respectively.

(iii) Final Probability: Computing the product of individual probabilities, as equation (4):

$$P\left(X|y\right)=\;\prod_{i=1}^{n}P\left(x_{i}|y\right).$$ (4)

(iv) Classification: Determining the class by comparing total probabilities, as equation (5):

$$P\left(Y|X\right)=\;\frac{P(Y)\prod_{i=1}^{n}P\left(x_{i}|Y\right)}{P(X)}.$$ (5)

This streamlined approach requires minimal training data, making naive bayes highly efficient for classification tasks.

#### 2.3.6. Artificial Neural Network (ANN).

ANNs are sophisticated predictive systems that perform exceptionally well when conventional statistical techniques are insufficient [51]. These networks excel at uncovering intricate linear and non-linear patterns within high-dimensional datasets and can manage non-linear variables [52]. ANNs consist of layers of nodes, input, hidden, and output, that simulate brain functions biologically [53]. The nodes in the input layer reflect the variables being evaluated, whereas the output layer corresponds to the categories. Due to their reliability in predicting outcomes and evaluating risk factors, ANNs have been successfully used in numerous medical applications, such as diagnostics, biochemical assessments, and drug discovery [54]. Their robust structure supports comprehensive data analysis and disease forecasting, making them essential in medical research.

#### 2.3.7. K-Nearest Neighbor Algorithm (KNN).

K-NN is a simple, non-parametric technique that classifies or predicts new data by evaluating the proximity of data points [55]. It selects the number K of closest neighbors and calculates the distance to all training points using Euclidean or Manhattan metrics [56]. The new data point is then classified by majority rule among its K nearest neighbors or predicted by averaging the outcomes in regression tasks [57]. This efficient method leverages spatial proximity, making it suitable for classification and regression without an underlying model.

#### 2.3.8. Adaptive Boosting (AdaBoost).

AdaBoost is a collective method to enhance prediction performance by combining several weak models into a stronger one [58]. It modifies the importance of misclassified samples in each cycle, allowing subsequent models to concentrate on rectifying prior errors [59]. The influence of each weak model is based on its error rate., and the overall model is progressively refined through this iterative approach, making AdaBoost effective for challenging classification problems [60].

#### 2.3.9. Gradient Boosting (GB).

GB is an ML approach that improves model accuracy by adding weak models sequentially [61]. Each subsequent model focuses on correcting the errors of the prior one by employing a differentiable loss function to guarantee ongoing refinement. This technique is widely used for enhancing classification and prediction results [62].

#### 2.3.10. Extreme Gradient Boosting (XGBoost).

XGBoost is an advanced gradient-boosting algorithm incorporating regularization methods to minimize overfitting [63]. It is recognized for its ability to scale, efficiency, and capacity to process numerical and categorical data. XGBoost utilizes parallel tree boosting to enhance differentiable loss functions, making it effective for tasks such as classification and regression [64]. Its robustness to outliers and flexibility in model tuning make it a preferred choice for advanced predictive analytics.

**Clustering algorithms**

#### 2.3.11. K-means.

The K-means clustering algorithm is a widely used technique for grouping data into clusters by following specific steps [65,66]:

- Randomly selecting a predetermined number of data points to serve as cluster centers.
- Assigning each data point to the cluster center closest to it.
- Calculating the average of each cluster’s points to establish new cluster centers.
- Repeating the process until the cluster assignments stabilize or a convergence criterion is met.

#### 2.3.12. Expectation Maximization (EM).

The EM algorithm performs the following four steps [67]:

(i) Estimation: The probability of each data point x belonging to a specific cluster $q_{i}$ is estimated based on the cluster’s mean $\left(m_{i}\right)$, standard deviation ${(s}_{i})$, and the cluster probability $\left(p_{i}\right)$. The basic equation for calculating the probability of each data point for a specific cluster. This can be seen in equation (6):

$$h_{i,n}=\left(\frac{p_{i}}{s_{i}}\right)e^{-\frac{1}{{2s_{i}}^{2\;}}{\left\|x_{n}-m_{i}\right\|}^{2}},$$ (6)

where ${\left\|x_{n}-m_{i}\right\|}^{2}$ is the squared distance between the data point and the cluster center.

(ii) Responsibility: The responsibility of each data point towards its cluster is calculated using the following formula, which considers the sum of the probabilities across all clusters, as outlined in equation (7):

$$h_{i,n}=\;\frac{(p_{i}/s_{i})e^{-\frac{1}{{2s_{i}}^{2\;}}{\left\|x_{n}-m_{i}\right\|}^{2}}}{\sum_{j}(p_{i}/s_{i})e^{-\frac{1}{{2s_{i}}^{2\;}}{\left\|x_{n}-m_{i}\right\|}^{2}}}.$$ (7)

(iii) Maximization: Based on the defined responsibility $h_{i,n}\;$for each data point towards cluster i, the mean (m) and standard deviation (s) for each cluster are recalculated. The new mean for each cluster is determined as presented in equation (8). The new center${\;m}_{i}$ is calculated as a weighted average of the data points, where the weights are the responsibilities $h_{i,n}\;$. Points with higher responsibility (that is, those closer to the cluster center) will have a greater influence on determining the new center.

$$m_{i}=\frac{\sum_{n}h_{i,n}x_{n}}{\sum_{n}h_{i,n}}.$$ (8)

(iv) Continuous Update: The process continues until the clusters stabilize or until a specified convergence criterion is achieved.

### 2.4. Hybrid machine learning techniques

The hybrid model methodology combines clustering and classification techniques to enhance predictive accuracy using a smaller set of Key Performance Indicators (KPIs). This approach integrates two main methods of hybridization [68].

#### 2.4.1. Sequential integration of classification and clustering.

This approach begins with either clustering or classification. In one method, clustering is applied first to segment the data and remove noise, resulting in representative clusters that feed into classification algorithms. This sequence helps train classifiers more effectively by focusing on cleaner data [69]. Alternatively, classification can be applied initially to categorize data, and the resulting labeled data informs further clustering, refining clusters based on category-specific characteristics [70].

#### 2.4.2. Combination of classification and clustering techniques.

This approach uses distinct classification or clustering algorithms in sequence. For example, one classifier refines the data by reducing noise, while a secondary classifier enhances performance by applying specific predictive rules [71]. Similarly, two clustering methods can be combined, where the first clustering algorithm reduces dataset size or dimensionality, making it easier for the second algorithm to achieve more precise clustering [65].

In addition to these strategies, ensemble techniques combine multiple classifiers, boosting predictive accuracy. This is done through majority or weighted voting, where each classifier’s output is considered to arrive at a consensus prediction, thereby reducing variance.

Overall, these hybridization approaches leverage the strengths of clustering and classification in identifying the most significant KPIs while retaining the predictive power. This methodology supports high accuracy in predicting HbA1c levels with fewer KPIs.

**Fig 1:**
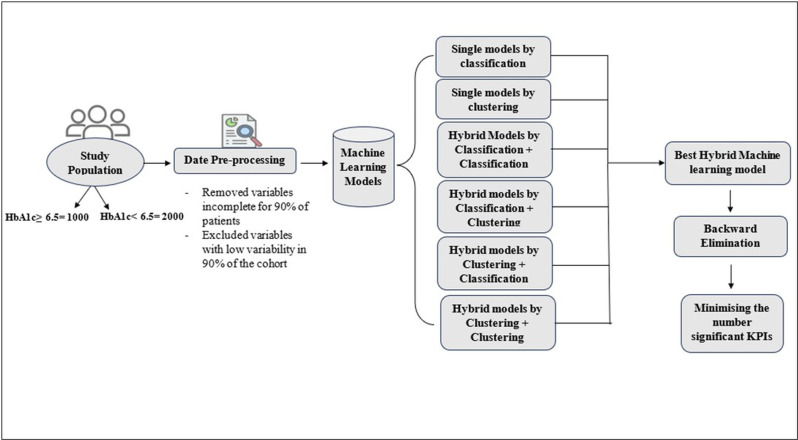
Architectural flow of hybrid models for data classification.

Fig 1 illustrates the data preparation process where incomplete variables for 90% of the patients or showed low variability across 90% of the sample are removed. Following this, the process starts with studying single models for classification or clustering, followed by hybrid models that combine classification and clustering techniques. After identifying the best hybrid machine learning model, the process progresses by applying backward elimination (BE) to reduce the number of significant KPIs. This step ensures that only the most impactful variables are retained to enhance the model’s efficiency and predictive accuracy in predicting HbA1c levels.

## 3. Performance evaluation metrics

To assess the models’ predictive performance, the dataset is divided, with 80% allocated for training and 20% for testing. Machine learning (ML) models are evaluated using six main metrics, including accuracy (ACC), precision (P), F-score, recall (R), the Receiver Operating Characteristic (ROC) curve, which represents the Area Under the Curve (AUC), and the Gini Index (Gini) [72,73].

The primary metric for evaluation is accuracy, which measures the proportion of correct predictions relative to the total predictions made. In the case of classifying patients based on HbA1c levels, accuracy is computed using the following equation:


$$Accuracy(ACC)=\;\frac{TP+TN}{TP+TN+FN+FP}$$

where *TP* (True Positives) represents correctly predicted HbA1c ≥ 6.5, *TN* (True Negatives) represents correctly predicted HbA1c < 6.5, *FP* (False Positives) indicates incorrectly predicted HbA1c ≥ 6.5 when it is HbA1c < 6.5, and *FN* (False Negatives) denotes incorrectly predicted HbA1c < 6.5 when it is HbA1c ≥ 6.5. These values are illustrated in Table 3.

**Table 3:** 2-Class confusion matrix.

	Predicted Positive	Predicted Negative
**Actual Positive**	True Positive (TP)	False Negative (FN)
**Actual Negative**	False Positive (FP)	True Negative (TN)

Precision is another important metric that focuses on the correct positive predictions. It is calculated using the equation:


$$Precision\;(P)=\;\frac{TP}{TP+FP}$$

Precision is the proportion of accurate positive predictions among all positive forecasts, which is important when false positives are costly.

On the other hand, recall assesses the model’s capacity to properly identify all true positive cases. Its value is calculated using the following equation:


$$Recall\;(R)=\frac{TP}{TP+FN}$$

Recall is particularly important in medical diagnostics predicting T2D, where missing a positive case (false negative) can have serious consequences.

Next, the F-score is used to balance precision (P) and recall (R), calculated as the harmonic mean of these two metrics:


$$F-score=\frac{2\times P\times R}{P+R}$$

The F-score provides a single metric that balances the trade-off between precision and recall, making it useful for evaluating models in imbalanced datasets.

The AUC offers a consolidated measure of the model’s performance by consolidating the ROC curve into one value. It is commonly used to assess the balance between sensitivity and specificity. However, the AUC has faced criticism for handling the false positive and false negative trade-offs. Furthermore, while the AUC effectively distinguishes between poor and strong models, it struggles to differentiate between strong models [74]. Additionally, the AUC has been criticized as a composite metric, with some researchers comparing it to integrating over a range of possible values, which they argue leads to its inconsistency as a performance measure [75]. The AUC can be computed as:


$$AUC=\int_{0}^{1}TPR(FPR)dFPR$$

where $TPR=\frac{TP}{TP+FN}$ (True Positive Rate) and $FPR=\frac{FP}{FP+TN}$ (False Positive Rate).

Moreover, the Gini Index is another metric derived from the AUC, and it is calculated using the following equation:


Gini=2×AUC−1

The Gini Index ranges from 0 to 1, where 1 indicates perfect discrimination between the classes, and 0 suggests no discriminative power.

## 4. Results

This section evaluates the performance of the recommended individual models (logistic regression (LR), decision tree (DT), random forest (RF), support vector machine (SVM), naive bayes (NB), artificial neural network (ANN), k-nearest neighbors (KNN), adaptive boosting (AdaBoost), gradient boosting (GB), extreme gradient boosting (XGBoost), k-means clustering (K-means), expectation-maximization (EM)), with four hybrid models formed by combining the best-performing models in classification and clustering. The first category, “classification + classification,” includes combinations of random forests with various classification algorithms. These include random forest + decision tree (RF + DT), random forest + naive bayes (RF + NB), random forest + logistic regression (RF + LR), random forest + artificial neural network (RF + ANN), random forest + support vector machine (RF + SVM), random forest + k-nearest neighbors (RF + KNN), random forest + random forest (RF + RF), random forest + adaptive boosting (RF + AdaBoost), random forest + gradient boosting (RF + GB), and random forest + extreme gradient boosting (RF + XGBoost). The second category, “classification + clustering” involves hybrid models combining random forest and clustering techniques. These combinations include random forest + expectation-maximization (RF + EM) and random forest + k-means clustering (RF + K-means). The third category, “clustering + classification” includes combinations of clustering algorithms, particularly k-means, with various classification techniques. These models are k-means + decision tree (K-means + DT), k-means + naive bayes (K-means + NB), k-means + logistic regression (K-means + LR), k-means + artificial neural network (K-means + ANN), k-means + random forest (K-means + RF), k-means + support vector machine (K-means + SVM), k-means + k-nearest neighbors (K-means + KNN), k-means + adaptive boosting (K-means + AdaBoost), k-means + gradient boosting (K-means + GB), and k-means + extreme gradient boosting (K-means + XGBoost). Lastly, in the “clustering + clustering” category, there are models that combine two clustering techniques. These combinations include k-means + expectation-maximization (K-means + EM) and k-means + k-means clustering (K-means + K-means), with independent variables detailed in Table 1.

The results indicate that the RF model achieved the highest accuracy of 0.87, precision of 0.88, recall of 0.87, F-score of 0.87, an AUC of 0.86, and Gini of 0.72 among other single models. In the comparison between clustering techniques K-means and EM, K-means (with K = 5) outperformed EM, attaining an accuracy of 0.63, precision of 0.70, recall of 0.69, F-score of 0.69, an AUC of 0.66, and Gini of 0.32. The best-performing model, RF, was used to create hybrid models in combination with nine other classification techniques. The hybrid model (RF + LR) outperformed the other hybrid models, with an accuracy of 0.88, precision of 0.88, recall of 0.88, F-score of 0.87, an AUC of 0.86, and Gini of 0.74, as shown in Table 4. The hybrid (K-means + RF) model outperformed other ‘Clustering + Classification’ models, achieving an accuracy of 0.81, a precision of 0.81, a recall of 0.62, F-score of 0.70, an AUC of 0.73, and Gini of 0.46. Furthermore, hybrid models combining ‘Classification + Clustering’ showed that the (RF + K-means) model achieved an accuracy of 0.62, a precision of 0.56, a recall of 0.62, F-score of 0.56, an AUC of 0.71, and Gini of 0.42. The hybrid (K-means + K-means) model was the best performing model in its category, achieving an accuracy of 0.62, a precision of 0.56, a recall of 0.62, F-score of 0.56, an AUC of 0.76, and Gini of 0.52, as shown in Table 4.

**Table 4:** Comparison of Model Performance based on KPIs listed in Table 1.

	Model	Accuracy	Precision	Recall	F-Score	AUC	Gini
Single models by classification	LR	0.86	0.84	0.85	0.85	0.85	0.70
	DT	0.81	0.81	0.81	0.80	0.81	0.62
	RF	**0.87**	**0.88**	**0.87**	**0.87**	**0.86**	**0.72**
	SVM	0.66	0.69	0.66	0.54	0.75	0.50
	NB	0.85	0.85	0.85	0.85	0.82	0.64
	ANN	0.67	0.72	0.67	0.68	0.73	0.46
	KNN	0.79	0.79	0.79	0.78	0.79	0.58
	AdaBoost	0.85	0.86	0.86	0.85	0.85	0.70
	GBoosting	0.85	0.85	0.85	0.85	0.85	0.70
	XGBoost	0.85	0.86	0.86	0.85	0.85	0.70
Single clustering Models	K-means	**0.63**	**0.70**	**0.69**	**0.69**	**0.66**	**0.32**
	EM	0.55	0.53	0.57	0.57	0.56	0.12
Hybrid Models by Classification + Classification	RF + DT	0.80	0.80	0.80	0.80	0.8	0.60
	RF + NB	0.85	0.85	0.85	0.85	0.85	0.70
	RF + LR	**0.88**	**0.88**	**0.88**	**0.87**	**0.87**	**0.74**
	RF + ANN	0.71	0.72	0.71	0.66	0.77	0.54
	RF + SVM	0.65	0.68	0.65	0.54	0.67	0.34
	RF + KNN	0.79	0.71	0.79	0.78	0.78	0.56
	RF + RF	0.85	0.85	0.85	0.85	0.85	0.70
	RF + AdaBoost	0.85	0.85	0.85	0.85	0.85	0.70
	RF + GBoosting	0.85	0.85	0.85	0.85	0.85	0.70
	RF + XGBoost	0.85	0.85	0.85	0.85	0.85	0.70
Hybrid models by Classification + Clustering	RF + EM	0.62	0.52	0.58	0.54	0.60	0.20
	RF + K-means	**0.62**	**0.56**	**0.62**	**0.56**	**0.73**	**0.46**
Hybrid models by Clustering + Classification	K-means + DT	0.80	0.77	0.68	0.72	0.78	0.56
	K-means + NB	0.81	0.83	0.71	0.76	0.82	0.64
	K-means + LR	0.81	0.82	0.73	0.77	0.76	0.52
	K-means + ANN	0.70	0.60	0.60	0.60	0.81	0.62
	K-means + RF	**0.81**	**0.81**	**0.62**	**0.70**	**0.71**	**0.42**
	K-means + SVM	0.65	0.75	0.56	0.55	0.78	0.56
	K-means + KNN	0.79	0.78	0.58	0.66	0.82	0.64
	K-means + AdaBoost	0.82	0.83	0.72	0.78	0.68	0.36
	K-means + GBoosting	0.82	0.84	0.72	0.78	0.59	0.18
	K-means + XGBoost	0.82	0.85	0.72	0.78	0.78	0.56
Hybrid models by Clustering + Clustering	K-means + EM	0.51	0.54	0.50	0.51	0.61	0.22
	K-means + K-means	**0.62**	**0.56**	**0.62**	**0.56**	**0.76**	**0.52**

**Fig 2:**
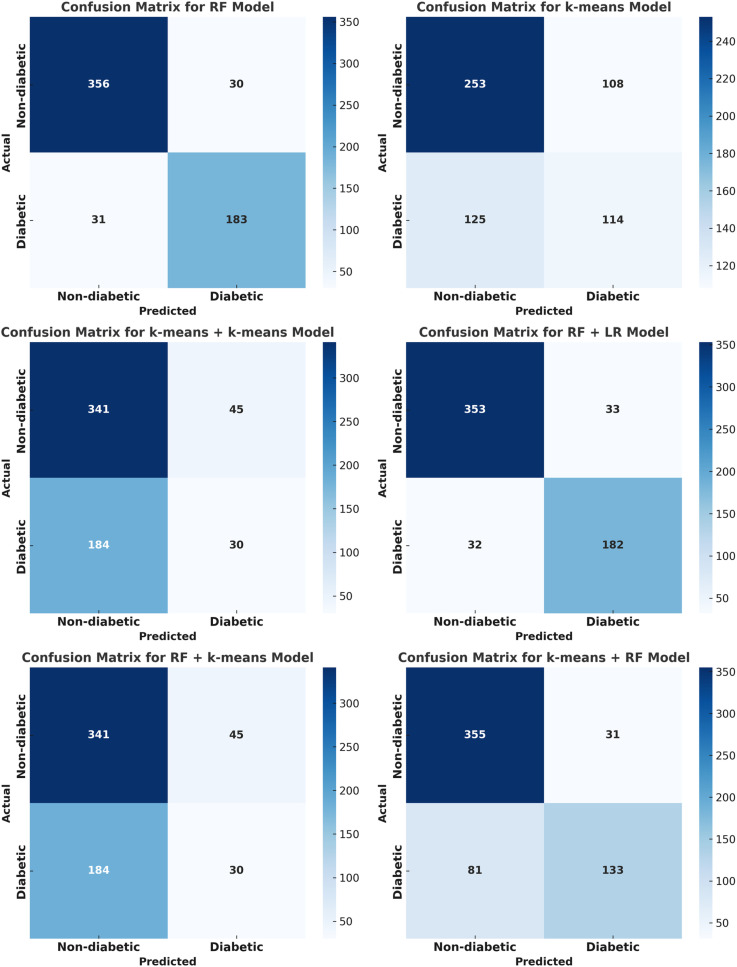
Confusion Matrix for the Hybrid algorithms.

Fig 2 displays confusion matrices for several models, including RF, (RF + LR), K-means, (K-means + K-means), (RF + K-means), and (K-means + RF). It illustrates true positives (TP), true negatives (TN), false positives (FP), and false negatives (FN) for diabetic and non-diabetic predictions.

**Fig 3:**
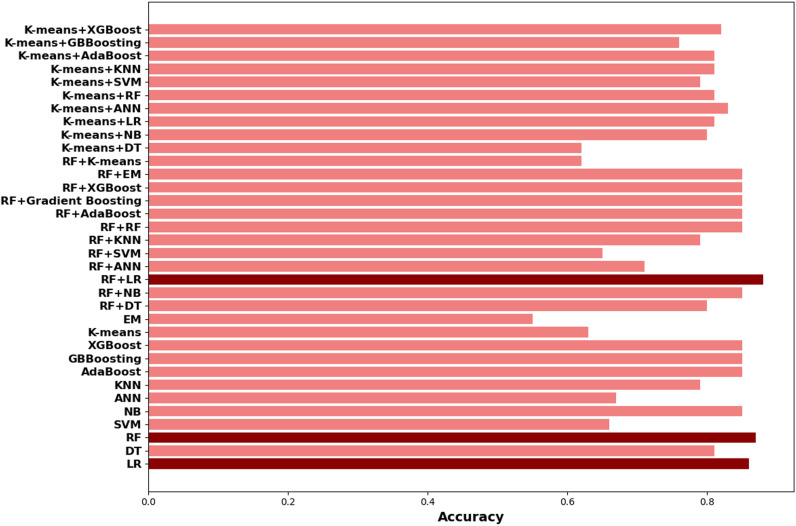
Comparison of Model Accuracy based on performance in Table 4.

Fig 3 illustrates the accuracy values for various machine learning models including both single and hybrid models. The hybrid models (RF + LR), RF, and LR demonstrate superior performance by achieving the highest accuracy (0.88, 0.87, 0.86), recall (0.88, 0.87, 0.85), precision (0.88, 0.88, 0.84), and F-Score (0.87, 0.87, 0.85), AUC (0.87,0.86,0.85), Gini (0.74, 0.72, 0.70), respectively. The figure allows for a comparison of model performance, highlighting the effectiveness of hybrid models in improving prediction accuracy.

## 5. Enhancing accuracy while minimising the number of significant KPIs

**Fig 4:**
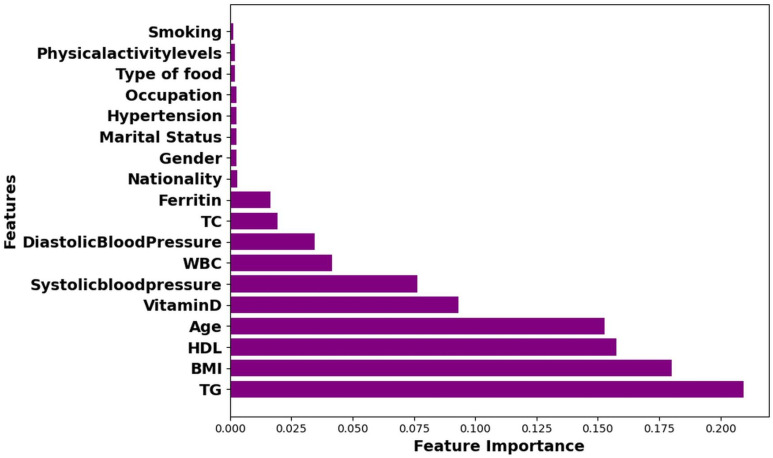
Ranking of Key Performance Indicators (KPIs) in RF model.

**Fig 5:**
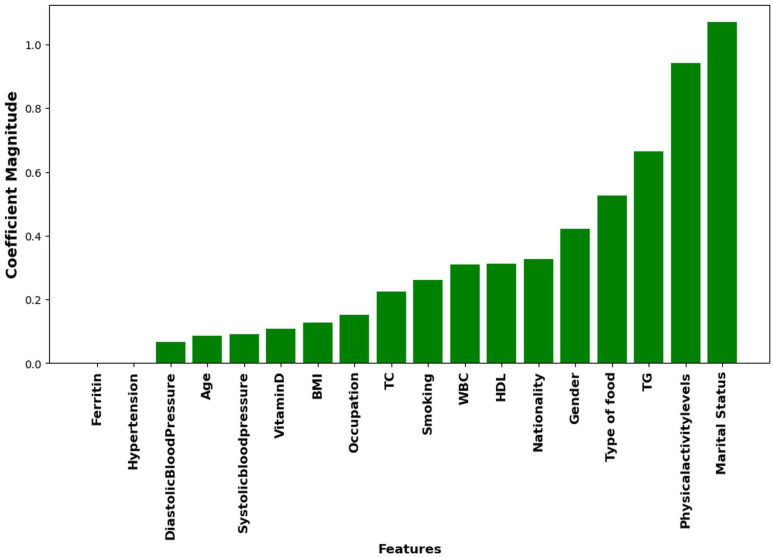
Ranking of Key Performance Indicators (KPIs) in LR model.

**Fig 6:**
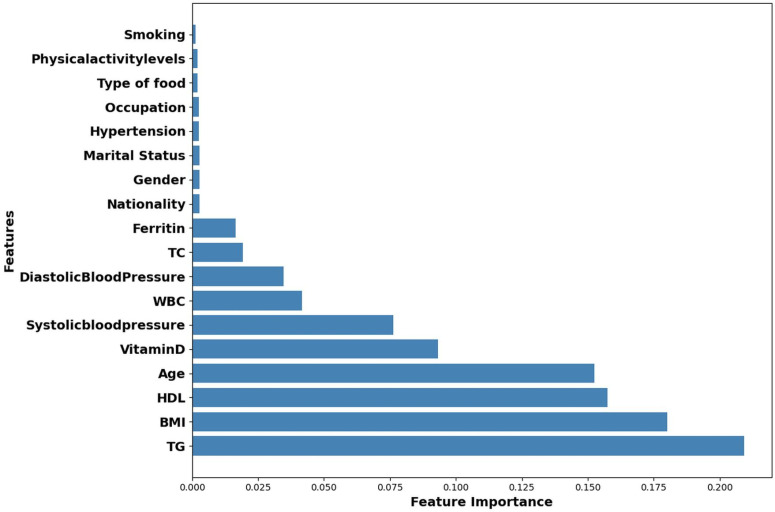
Ranking of Key Performance Indicators (KPIs) in Hybrid (RF + LR) model.

This research aims to deploy hybrid models to improve the prediction accuracy of HbA1c while reducing the number of independent predictors associated with T2D. Reducing the number of significant KPIs will enable health practitioners to identify those at risk of developing T2D by monitoring a smaller number of KPIs. Figs 4–6 highlight the top ten KPIs that significantly impact the prediction accuracy of RF, LR, and Hybrid (RF + LR) models. These variables are DBP, WBC, SBP, vitamin D, ferritin, age, HDL, BMI, TC, and TG. A backward elimination (BE) method was applied at each classification step to remove the least important KPIs, aiming to retain only the most impactful ones.

Table 5 illustrates the difference in accuracy among different models, Hybrid (RF + LR), RF, and LR, as the number of KPIs is gradually minimized. Both the hybrid (RF + LR) and RF models achieved a high accuracy of 0.95 when using all ten KPIs. However, as the number of KPIs was reduced through backward elimination (BE), the accuracy of the hybrid model remained relatively stable until only four KPIs (Age, BMI, HDL, and TG) were left. The hybrid model achieved an accuracy of 0.93, precision of 0.95, recall of 0.90, F-score of 0.92, an AUC of 0.88, and Gini of 0.76 using these 4 KPIs compared with an accuracy of 0.95, precision of 0.97, recall of 0.92, F-score of 0.94, an AUC of 0.96, and Gini of 0.92 based on 10 KPIs. RF achieved an accuracy of 0.95 using 10 KPIs, which gradually decreased as the number of KPIs decreased. Its accuracy dropped to 0.81 when only 4 KPIs were used. Moreover, age, BMI, HDL, and TG were identified as critical factors, as shown in Figs 4–6. However, when one of these features was removed, the hybrid model’s accuracy dropped significantly to 0.85, highlighting the importance of these four KPIs in maintaining the model’s predictive accuracy. The results presented in Table 5 underscore the ability of hybrid models to enhance the accuracy of predicting HbA1c while the number of KPIs is reduced from 10 to only 4.

**Table 5:** Accuracy of RF, LR, and hybrid (RF + LR) Models by KPIs Count.

No. of KPIs	LR						RF						Hybrid (RF + LR)					
	Accuracy	Precision	Recall	F-score	AUC	Gini	Accuracy	Precision	Recall	F-score	AUC	Gini	Accuracy	Precision	Recall	F-score	AUC	Gini
10	0.87	0.89	0.84	0.86	0.85	0.7	0.95	0.97	0.92	0.94	0.93	0.86	0.95	0.97	0.92	0.94	0.96	0.92
9	0.76	0.78	0.73	0.75	0.74	0.48	0.95	0.97	0.92	0.94	0.93	0.86	0.95	0.97	0.92	0.94	0.94	0.88
8	0.75	0.77	0.72	0.74	0.73	0.46	0.94	0.96	0.91	0.93	0.92	0.84	0.95	0.97	0.92	0.94	0.93	0.86
7	0.75	0.77	0.72	0.74	0.73	0.46	0.92	0.94	0.89	0.91	0.9	0.80	0.94	0.96	0.91	0.93	0.92	0.84
6	0.75	0.77	0.72	0.74	0.75	0.5	0.91	0.93	0.88	0.9	0.89	0.78	0.94	0.96	0.91	0.93	0.91	0.82
5	0.77	0.79	0.74	0.76	0.72	0.44	0.9	0.92	0.87	0.89	0.88	0.76	0.94	0.95	0.9	0.92	0.91	0.82
4	0.73	0.75	0.7	0.72	0.72	0.44	0.81	0.83	0.78	0.8	0.83	0.66	**0.93**	0.95	0.9	0.92	0.88	0.76
3	0.73	0.75	0.7	0.72	0.70	0.40	0.77	0.79	0.74	0.76	0.79	0.58	0.85	0.87	0.82	0.84	0.80	0.60
2	0.71	0.73	0.68	0.7	0.69	0.38	0.71	0.73	0.68	0.7	0.75	0.50	0.82	0.84	0.79	0.81	0.78	0.56
1	0.62	0.64	0.59	0.61	0.60	0.20	0.61	0.66	0.61	0.63	0.63	0.26	0.74	0.76	0.71	0.73	0.68	0.36

**Fig 7:**
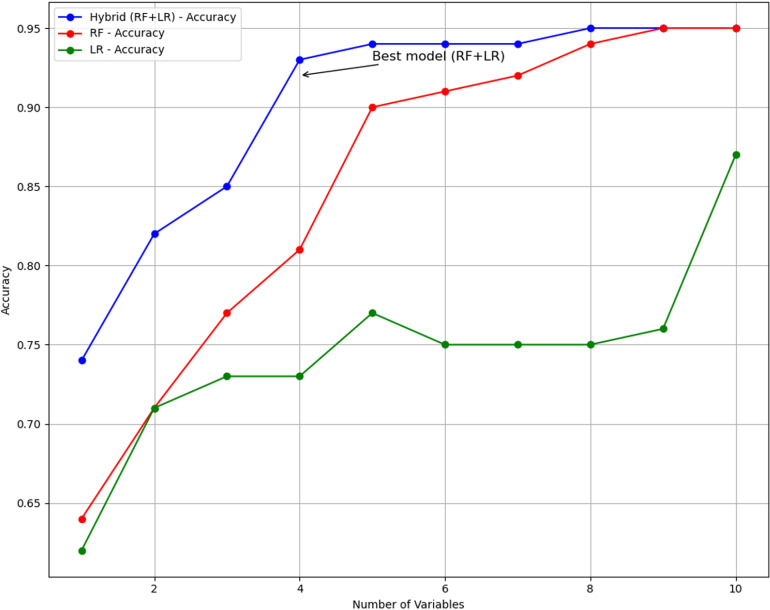
Comparison of Model Accuracy (Hybrid (RF + LR), RF, and LR) across important KPIs.

Fig 7 compares the accuracy of three models, the hybrid (RF + LR), RF, and LR, across important KPIs. The hybrid model (RF + LR) consistently demonstrates higher accuracy, especially when the number of variables is reduced to only 4 KPIs, highlighting its superior performance. The RF model shows a gradual decrease in accuracy as the number of variables decreases. In contrast, the accuracy of the LR model starts high but decreases significantly as the number of variables is reduced.

**Fig 8:**
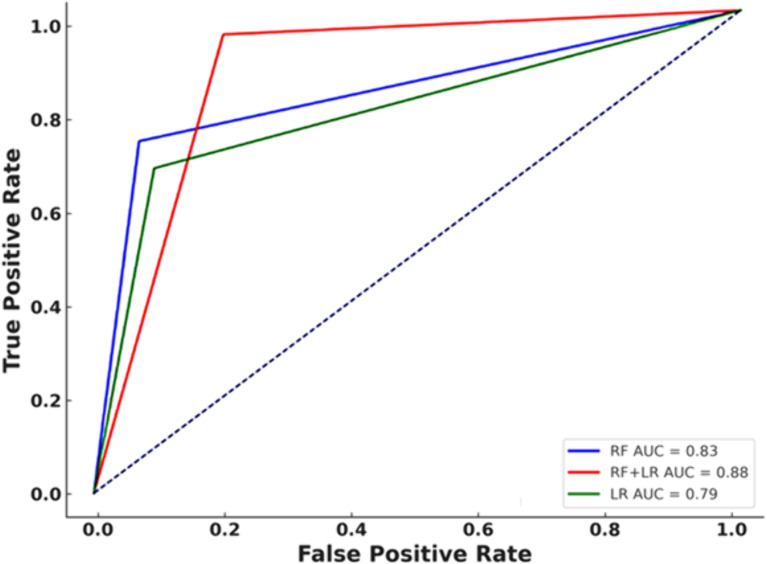
ROC curves for model performance comparison.

The ROC curves for the top three predicting models are demonstrated in Fig 8 (using 4 KPIs). it shows that the hybrid model (RF + LR), which achieved an AUC of 0.88, outperforms other models.

## 6. Discussion

As far as we know, this study is the first to investigate the relationship between lifestyle factors, demographic characteristics, and lipid profiles with T2D in Saudi Arabia using hybrid methods. Local case-control data were used to create a clinically relevant and highly accurate hybrid predictive model for HbA1c levels and reduce the number of key associated predictors. To guarantee extensive coverage of the heterogeneous community, this study utilised anonymised data from 3,000 patients aged 20 and above at King Abdulaziz University Hospital (KAUH) in Saudi Arabia. The data was analyzed to classify participants into two groups: the case group (n = 1000) with elevated HbA1c values (≥ 6.5) and the control group (n = 2000) with normal levels (< 6.5).

We applied several hybrid machine learning models, including “Classification + Classification”, “Classification + Clustering”, “Clustering + Clustering”, and “Clustering + Classification” to enhance prediction accuracy. The performance of these models in predicting HbA1c levels was compared using accuracy, precision, recall, F-score, and AUC. The findings, as detailed in Table 4, utilizing all variables listed in Table 1, indicate that the hybrid model combining RF and LR and the single model RF demonstrated outstanding performance, achieving accuracies of 0.88 and 0.87, respectively. Additionally, both models recorded precisions of 0.88, recall rates of 0.88 and 0.87, F-scores of 0.87, an AUC of 0.87 and 0.86, and Gini of 0.74 and 0.72 each.

We then employed the top 3 performing models, hybrid (RF + LR), RF, and LR, from Table 5, together with the top ten significant variables identified by these models Figs 4–6, to enhance their prediction accuracy while reducing the number of KPIs through the backward elimination (BE) process. The significant group of variables associated with the HbA1c level is determined by the subset with the highest accuracy or close to the highest accuracy when the number of KPIs was reduced from 10 to 4.

The result presented in Table 5 shows that the hybrid accuracy was almost the same even when the number of KPIs was reduced from 10 to only 4 (0.95 (KPIs = 10), and 0.93 (KPIs = 4)), as shown in Fig 7. However, there was a significant decrease in the prediction accuracy of RF and LR (RF 0.95 vs 0.81 and LR 0.87 vs 0.73). Notably, the hybrid combination of the RF and LR models demonstrated robust performance across several metrics: it achieved an accuracy of 0.93, with precision of 0.95, recall of 0.90, F-score all at 0.92, an AUC of 0.88, and Gini of 0.76 when the number of KPIs was reduced from ten to four: age, BMI, HDL, and TG. Using fewer KPIs may assist healthcare providers in monitoring and managing the most significant factors influencing HbA1c levels more effectively.

The findings indicated that elevated HbA1c levels are more prevalent among elderly individuals who suffer from obesity, higher TG levels, and low HDL, as reported in Table 2. These results contradict the findings in references [76,77] despite being consistent with previous studies cited in [78,79]. The differences may be due to variations in study design and the countries where the research was conducted. Obesity is a prominent modifiable risk factor for T2D. Our results showed that 45.7% of participants had a body mass index exceeding the normal range, as highlighted in Table 1. The high rate of obesity in Saudi Arabia is linked to several factors, including a diet high in fats and calories, with 64.9% of participants reporting frequent consumption of unhealthy foods. Based on Table 1, the results showed that the cases suffer from low levels of HDL and elevated levels of TG, which can be attributed to the unhealthy dietary habits prevalent in Saudi society.

**Fig 9:**
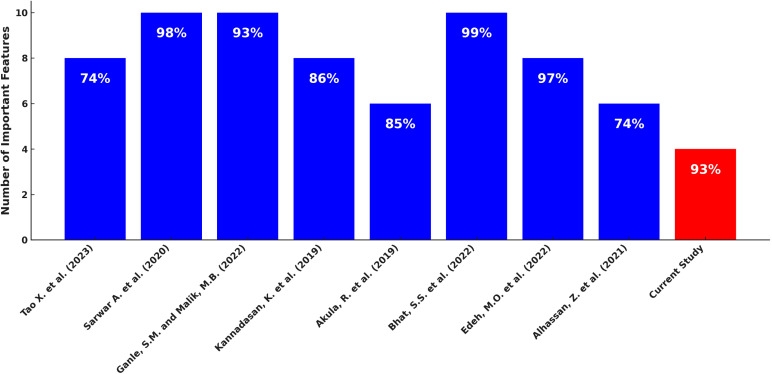
Accuracy comparison of the proposed hybrid and existing model’s vs the number of KPIs in individual models.

We have also compared the accuracy of the existing predictive models with our proposed hybrid (RF + LR), focusing on the number of significant KPIs used in their models. The results presented in Fig 9 show that previous studies used more variables to achieve high predictive accuracy. For instance [26], used nine variables to achieve an accuracy of 74.6%, [31] employed ten variables to reach an accuracy of 98% [80], utilized ten variables to attain an accuracy of 93% [81], used eight variables to achieve an accuracy of 86.26% [29], achieved an accuracy of 85% with six variables [32], employed ten variables to reach an accuracy of 99%. Authors in [82] employed eight variables and achieved an accuracy of 97%, and [20] used six variables to achieve an accuracy of 74.55%, as shown in Fig 9.

In contrast, our proposed hybrid model, combining random forest and logistic regression (RF + LR), achieved an accuracy of 93% using only four KPIs.

## 7. Conclusions

Type 2 diabetes (T2D) is a chronic health condition and a significant global public health challenge, imposing both economic and social burdens. It is a major concern for healthcare systems, policymakers, medical professionals, and individuals affected by the disease. Saudi Arabia currently ranks seventh worldwide in terms of prevalence rate. However, despite this high rate, studies focused on T2D in the country remain limited compared to those conducted in developed nations.

Existing research on T2D in Saudi Arabia is limited to a small number of contributing factors. This research represents the case-control analysis exploring the relationship between demographic, lifestyle, and lipid profile factors and the risk of developing T2D in the Saudi Arabian population aged between 20 and 99. We have utilised case-control data from 3,000 medical records obtained from King Abdulaziz University Hospital. The data includes a broad array of demographic, lifestyle, and lipid profile key indicators identified in scientific literature to enhance the comprehensiveness of the analysis for patients.

It is well documented that the HbA1c level is widely regarded as the most dependable marker for T2D. For the first time, we explored multiple approaches to identify the optimal model to predict the HbA1c level and identify the most significant KPIs associated with it, leveraging various hybrid techniques in response to the increasing interest in their application within the healthcare sector. The efficacy of the models was assessed using Precision, Recall, F-score, Area Under the Curve (AUC), and Gini Index (Gini).

We developed hybrid models by combining two single models (classification and clustering) to enhance the predictive accuracy of HbA1c levels using local data. This is followed by using a backward elimination approach to reduce the number of significant factors in the top-performing models while assessing their predictive accuracy.

The significant group of variables associated with the HbA1c level is identified by the subset that achieves the highest accuracy or close to the highest accuracy while utilising fewer variables. The results show that the proposed hybrid model combining LR and RF attains the highest accuracy of 93% with the smallest subset of 4 variables. In comparison, the LR model achieves an accuracy of 87% with ten variables, while the RF model reaches an accuracy of 92% with seven variables. The significant variables identified by the proposed model through the backward elimination approach are age, body mass index (BMI), triglycerides (TG), and high-density lipoprotein (HDL).

The analysis of HbA1c-related factors revealed differences between the case and control groups. The average age in the case group was 62 years compared to 70 years in the control group. There was a notable increase in BMI in the case group, accompanied by lower levels of HDL and triglycerides (TG), as shown in Table 1.

This study highlights the importance of utilising hybrid models as an effective tool for accurately predicting HbA1c levels to identify those at risk of developing T2D monitoring smaller number of KPIs. The results presented in this paper significantly contribute to T2D research in Saudi Arabia.

The proposed hybrid model aids healthcare professionals by improving their ability to make accurate, data-informed clinical decisions in a timely manner, while considerably decreasing the number of necessary indicators. This enables them to concentrate on the most significant risk factors, thereby promoting timely and data-driven clinical decision-making.

## 8. Limitations

This study offers a significant contribution by developing a hybrid model (RF + LR) to enhance HbA1c predictive accuracy with a minimal number of predictors. However, a few limitations should be considered for future improvements. First, the data utilised in this study was collected from a single medical institution. While the relatively large and diverse sample may support the internal validity of the findings, the inclusion of nationwide data will improve the sample size and diversity of the results and ensure its validity at the national level. Second, feature selection was performed using backward elimination. While this method helps reduce the number of predictors, combining it with domain expertise or regularization-based approaches such as LASSO regression could further enhance the model’s reliability and interpretability. Third, the hybrid approach in this study combined only two algorithms at a time. Future studies could explore the combination of more than two algorithms as well as, including more advanced ensemble models or deep learning techniques to further enhance accuracy and flexibility. Fourth, additional variables such as medical history, genetic factors, or psychological factors could be added, which may affect HbA1c levels. Fifth, the absence of a nationwide health database limited the variety and representativeness of the data. Developing a national system that links hospitals across different regions would provide better data diversity and support stronger, more general models for diabetes prediction in Saudi Arabia.
